# Successful Transabdominal Removal of Penetrating Iron Rod in the Rectum: A Case Report

**DOI:** 10.24248/eahrj.v5i2.663

**Published:** 2021-11-15

**Authors:** Jay Lodhia, David Msuya, Kondo Chilonga, Danson Makanga

**Affiliations:** a Department of General Surgery, Kilimanjaro Christian Medical Center, Moshi Tanzania; b Kilimanjaro Christian Medical University College, Moshi Tanzania; c Department of General Surgery, Mpeketoni Hospital, Lamu County, Kenya

## Abstract

Foreign bodies in the anus and rectum are not uncommon presentations globally. Reasons for foreign bodies in the rectum can be trauma, assault, psychiatric reasons but the most common reason documented is sexual pleasure, and objects range from sex toys to tools to packed drugs. Regardless of the reason, health care providers must maintain nonjudgmental composure and express empathy.

Numerous cases have been reported of anorectal foreign body due to various causes. Removal of the objects has mostly been through rectally but some does need surgical intervention. A multidisciplinary approach and radiologic investigations are important to guide in the management outline. Establishment of guidelines for anorectal foreign bodies are needed to guide surgeons and emergency physicians on the course of treatment.

We present a case of an eleven-year old school boy slid and fell on an iron rod that penetrated his rectum through his anal canal. Presented with clinical features of peritonitis, where emergency laparotomy was done and the iron rod was extracted abdominally with primary repair of the rectum. The boy recovered well and was discharged four days after with no complications.

## INTRODUCTION

The earliest cases of anorectal trauma due to foreign body reports go back to the 1500s and since then the numbers have increased of reports of foreign body in the rectum^1,2^. Many objects have been mentioned to have been found in the lower gastrointestinal tract including screw drivers and even a tool kit, but the most common reason for anal insertion by far is said to be sexual plessure^1,3^. Occasionally some objects have been ingested and are passed through the entire gastrointestinal tract and be lodged in the rectum.^2,4^ Patients usually are reluctant to seek medical attention as it may be an embarrassing situation therefore it is important for clinicians to maintain compassion and nonjudgmental composure^1^.

Penetrating injuries in the anorectal region in children are rare but when encountered they have high morbidity and mortality^5^. In young patients however, the incidence is rising due to auto-erotic acts and behavior disorders^3^. Generally colorectal trauma is mainly encountered in the military setting at 5-10% compared to civilian setting at 1-3% incidence^6^. It is essential in the management to have a multidisciplinary approach, from the paramedic surgeons and also the fire department, as they have the appropriate tools to cut the and free the object to aid transportation and removal^3,5^. There are many endoscopic and surgical techniques on literature described on removal of various foreign bodies from the anorectal. We report the successful extraction of an iron rod that penetrated the rectum through the anus in an 11-year old school boy.

## CASE PRESENTATION

**FIGURE 1:**
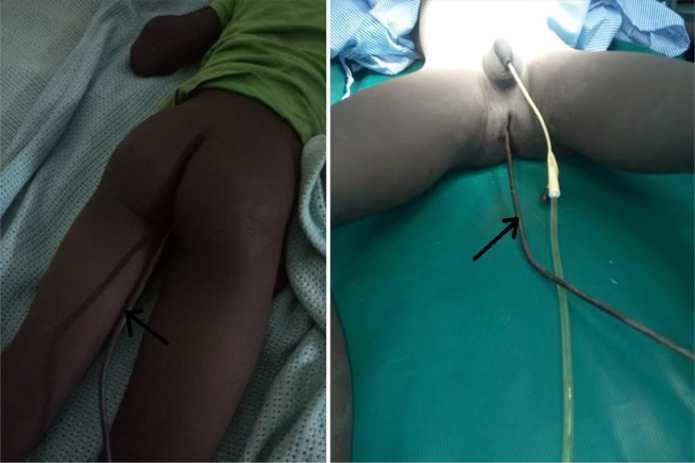
Iron Rod Lodged into Rectum through Anal Orifice

**FIGURE 2:**
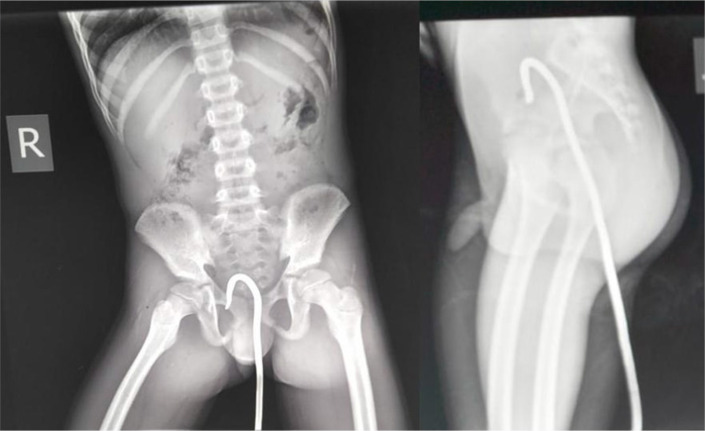
X-Ray Showing Metallic Object with Curved End into Rectum with No Emphysema

An 11-year-old boy presented to us after falling accidentally on a metal rod six hours after the incident. He was walking home from school, slid and fell on a metal rod with a curved end in an abandoned construction site. He was brought to the hospital in a private car by his mother directly after the incident. The iron rod went through his anal orifice. The child reported of some lower abdominal pain that was accompanied by some fresh bleeding from the rectum. The child denied urine incontinence. Upon examination he was a well nourished child, fully alert, in moderate pain, mild conjunctival pallor, saturating at 95% on room air, pulse rate of 104 beats per minute, blood pressure of 98/65 mmHg and had axillary temperature of 38°C. On local examination, there were no perianal injuries noted with normal anal verge and muscle tone but a rod into the anal orifice (Figure 1). His abdomen moved with respiration, had rebound tenderness and guarding, with reduced bowel sounds. Plain abdominal pelvic x-rays were done showing the rod in the rectum with no surrounding emphysema (Figure 2). Our working diagnosis was peritonitis due to perforated bowel, and was scheduled for an emergency laparotomy. The boy was given tetanus toxoid, intravenous Ceftriaxone, Metronidazole and Paracetamol as pre and post operative antibiotics.

**FIGURE 3.**
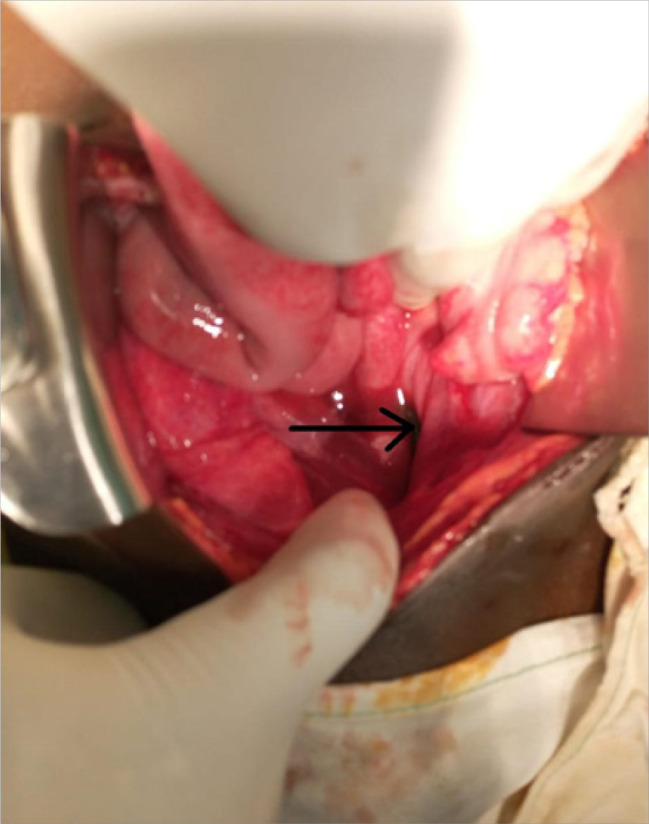
Curved End of the Iron Rod Perforated Anterior Wall of Rectum

**FIGURE 4:**
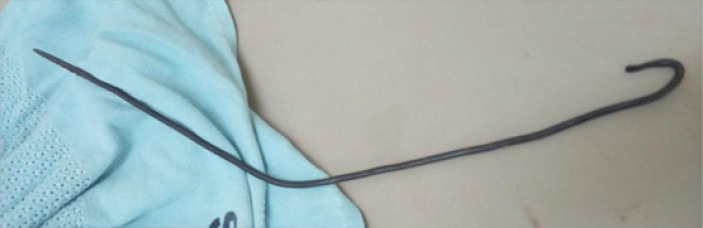
Iron Rod with Curved End (Approximately 100 Cm Long and 400 Grams)

Intra-operatively, through a midline incision, there was turbid ascites that was not foul smelling. The iron rod hook noted in the abdominal cavity having perforated the anterior part of the rectum at the peritoneal reflection (Figure 3). The rod was extracted abdominally, refreshening and primary closure done of the rectum. The iron rod was hooked at one end, approximately a meter long, weighed about 400 grams and did not have rust on its surface (Figure 4). The boy fared well post operatively and was nursed in the general ward for four days before being discharged, as his vitals were within range with no complications encountered during the operation. During his stay there was no signs and symptoms of local and systemic infection and was passing stools normally per rectally. He was then reviewed at the outpatient unit three weeks later whereby his abdominal incision had healed, did not have any abdominal signs or symptoms and had resumed his normal activities including schooling.

## DISCUSSION

Many patients with rectal foreign bodies are men aged between 20 and 40 years, and present to the hospital after exhausting all the efforts of removing the object at home^2,3^. The most common causes are behaviour disorders and sexual pleasure in recent years^3^. The true incidence is not known of foreign bodies in the rectum as many do not seek medical attention for obvious reasons. Objects are sometimes inserted involuntarily and therefore require extra care as they are often cases of abuse or rape and especially in children^3^. Whatever the cause, the treatment of these patients require a multidisciplinary approach to avoid serious complications^3,5^.

There are various methods mentioned by Gentile et al. on removal of the foreign bodies, and they include manual trans anal extraction under sedation, laparoscopic assisted trans anal extraction and laparotomy being the last resort. The authors also mention the attempt to milk the foreign body out during laparotomy even if the gastrointestinal tract is perforated^3^. Similarly, Gajjar et al. also state the importance of trans anal removal of the foreign object during a laparotomy with closure of the perforationwith a diversion colostomy. The authors also highlight the importance of colonoscopy and abdominal x-ray to rule out injury after removal^2^. A colotomy can be made if the object cannot be pushed out, and colostomy if the peritoneum is contaminated from a perforation^3^.

In our case, the rod had perforated the anterior rectum with minimal peritoneal contamination hence the object was abdominally removed and primary repair was done of the perforation successfully. This could also be because the patient presented early and surgery was done early resulted in successful outcome unlike the case reported by Shaban et al where their patient had a foreign body for a week and also perforated the rectum^1^.

Shaban et al continue to mention that the preferred and first line management of foreign bodies in the rectum is conservative followed by minimal invasive and lastly surgery with or without colostomy if the gastrointestinal tract has been perforated. They state that primary repair can be done if the patient has limited injury (lacerations less than 50% of the circumference) which was in our case, however the surgeon should decide intra-operatively depending on the contamination of the peritoneal cavity^1^.

In a similar case report by Ozaydin et al, the authors highlight the importance of early intervention for improved outcome in terms of sepsis control and early wound healing, as this was the pathway of management in our case^5^. A French study by Goin et al studied the feasibility of non-operative management (NOM) for treatment of penetrating abdominal trauma, in which the failure rate was 7.2% and the authors concluded that NOM is safe for trauma patients, reduced hospital stay and cost nevertheless CT-scan can aid in patient selection^7^.

Another similar study also showed the laparoscopic surgery in abdominal trauma patients has less postoperative pain, lesser wound infection, short hospital stays and comparing to laparotomy neither had missed injuries^8^. Proximal fecal diversion by colostomy is the most conservative management for extraperitoneal rectal trauma due to the difficulty in mobilization and anastomosis. In contrast, intraperitoneal rectal injuries can be repaired primarily with or without diversion depending on the surgeon's discretion. Distal rectal wounds could be repaired if accessible transanally^9^.

A different study showed that those with intraperitoneal injury managed with fecal diversion developed more abdominal complications (p=.003) and concluded that most patients with intraperitoneal injuries underwent direct repair or resection whereas diversion did not improve the outcomes^10^. As in the index case, the colon was primarily repaired as the patient did not have a high risk of anastomotic leak and the nature of injury was non-destructive as described by Brown et al^11^.

Management of penetrating trauma to the rectum is still not clear globally, but key principles are primary closure, fecal diversion or distal rectal washout as there is limited evidence on this area and needs to be further explored for possible international guidelines^8,11^.

## CONCLUSION

Minimal invasive surgery with removal of the foreign body per anus should be the management of choice under sedation or general anesthesia to avoid iatrogenic trauma and also comfort for the surgeon. Rectal examination is equally important in the initial examination along with radiological investigations to know the characteristics of the foreign body and its relation to the surrounding soft tissue of the patient.

The ability to do primary repair of the injured large bowel depends on the analysis of the operating surgeon, depending on the severity of the tissue injury, amount of contamination and the time of presentation from the initial incident, though controversies do exist as no specific guidelines have been established for removal of rectal foreign body.

## References

[1] Shaban Y, Elkbuli A, Ovakimyan V, Wobing R, Boneva D, McKenney M, Hai S. Rectal foreign body causing perforation: Case report and literature review. Annals of Medicine and Surgery. 2019 Nov 1; 47:66-9. 31645940 10.1016/j.amsu.2019.10.005 PMC6804320

[2] Gajjar RA, Gupta PB. Foreign body in the rectum: A challenge for the emergency physician. Journal of family medicine and primary care. 2016 Apr;5(2):495. 10.4103/2249-4863.192381 PMC5084595 27843875

[3] Gentile M, Cestaro G, Di Filippo G, Amato B, Sivero L. Successful transanal removal of unusual foreign body self-inserted in the rectum. Ann. Ital. Chir. 2019;90(1):88-92. 30737362

[4] Ye H, Huang S, Zhou Q, Yu J, Xi C, Cao L, Wang P, Gong Z. Migration of a foreign body to the rectum: a case report and literature review. Medicine. 2018 Jul;97(28). 10.1097/MD.0000000000011512 PMC6076194 29995819

[5] Ozaydin S, Gulleroglu A, Karaaslan B, Celebi S, Besik C, Toker MK, Sander S. Penetrating injury caused by a long iron bar: A case report. Northern clinics of Istanbul. 2018;5(1):75. 29607439 10.14744/nci.2017.75508 PMC5864715

[6] Brown CV. Penetrating injuries to the colon and rectum. Current Trauma Reports. 2015 Jun 1;1(2):113-8.

[7] Goin G, Massalou D, Bege T, et al. Feasibility of selective non-operative management for penetrating abdominal trauma in France. Journal of visceral surgery. 2017 Jun 1;154(3):167-74. 27856172 10.1016/j.jviscsurg.2016.08.006

[8] Lim KH, Chung BS, Kim JY, et al. Laparoscopic surgery in abdominal trauma: a single center review of a 7-year experience. World Journal of Emergency Surgery. 2015 Dec 1;10(1):16. 26056529 10.1186/s13017-015-0007-8 PMC4459684

[9] Wu K, Posluszny JA, Branch J, et al. Trauma to the pelvis: Injuries to the rectum and genitourinary organs. Current Trauma Reports. 2015 Mar 1;1(1):8-15.

[10] Brown CV, Teixeira PG, Furay E, et al. Contemporary management of rectal injuries at level I trauma centers: the results of an American Association for the Surgery of Trauma multi-institutional study. Journal of Trauma and Acute Care Surgery. 2018 Feb 1;84(2):225-33. 10.1097/TA.0000000000001739 29140953

[11] Ahern DP, Kelly ME, Courtney D, et al. The management of penetrating rectal and anal trauma: a systematic review. Injury. 2017 Jun 1;48(6):1133-8. 28292518 10.1016/j.injury.2017.03.002

